# Fluorescence of Intrinsic Milk Chromophores as a Novel Verification Method of UV-C Treatment of Milk

**DOI:** 10.3390/foods13182887

**Published:** 2024-09-12

**Authors:** Kallis Souroullas, Andreas Manoli, Grigorios Itskos, Theofylaktos Apostolou, Photis Papademas

**Affiliations:** 1 Department of Agricultural Sciences, Biotechnology and Food Science, Cyprus University of Technology, Limassol 3036, Cyprus; kc.souroullas@edu.cut.ac.cy; 2 Experimental Condensed Matter Physics Laboratory, Department of Physics, University of Cyprus, Nicosia 1678, Cyprus; manoli.g.andreas@ucy.ac.cy (A.M.); itskos@ucy.ac.cy (G.I.); 3 EMBIO Diagnostics Ltd., Nicosia 2018, Cyprus; theo.apo@embiodiagnostics.eu

**Keywords:** UV-C, fluorescence spectroscopy, photooxidation, chromophores, riboflavin, intrinsic biomarkers, milk preservation, non-thermal processing, right-angle fluorescence, front-face fluorescence

## Abstract

The European Food Safety Authority (EFSA) has approved the use of a 1045 J/L UV-C dose as an adjunct to pasteurization to increase the shelf life and vitamin D3 content of milk. However, there are no verification methods analogous to the alkaline phosphatase test for pasteurized milk to ensure that the desired UV-C dose has been correctly applied. The aim is to develop a real-time in-line detector based on fluorescence spectroscopy. In this study, 22 different UV-C doses (ranging from 0 to 2000 J/L) were applied to milk to assess the impact of photooxidation on intrinsic photosensitive chromophores. Fluorescence spectroscopy (90°-angle) was employed as the method of analysis for monitoring the changes in the fluorescence spectra of chromophores in milk without sample pretreatment. Three important chromophore areas (CAs) were identified: CA 1 (riboflavin), CA 3 (vitamin A and dityrosine) and CA 4 (tryptophan), with statistically significant changes at around 1045 J/L and 1500 J/L. The findings of our preliminary study support our hypothesis that the fluorescence of intrinsic chromophores can be used as verification of the applied UV-C dose.

## 1. Introduction

UV-C processing units (~254 nm) are recognized for their ability to efficiently inactivate pathogens and spoilage microorganisms in milk (dairy) and other foods by inducing DNA damage in microbes and eventually cell death, leaving no chemical residues and maintaining nutritional quality. This processing method is gaining attention as an environmentally friendly and cost-effective method with low maintenance, low installation costs and minimal energy use [1]. The low operational cost of UV-C processing, ranging from $0.01 to $0.05/L (as opposed to thermal pasteurization, which ranges from $0.03/L to $0.08/L) coupled with the environmental and financial benefits of UV-C over conventional technologies, suggests favorable market potential. The market for UV disinfection equipment, valued at $1.3 billion in 2019, is expected to rise to $5.7 billion by 2027, indicating a significant growth trend [2,3]. 

Countries that have approved the use of UV-C for milk treatment include the United States, the European Union, Ireland, Israel, Canada, and India [2]. The EFSA has reviewed the safety of milk processed with UV-C light and has found it to be safe within the proposed usage conditions provided by the applicant, i.e., the application of 1045 J/L of UV-C dose as an adjunct of pasteurization. This assessment applies to all types of cow’s milk (whether whole, semi-skimmed, or skimmed) that is treated with UV-C light after undergoing pasteurization to extend the product’s shelf life and vitamin D3 content [4]. 

Currently the process conditions of the UVC reactor act as the only verification step of the Critical Control Point (CCP); i.e., to verify the CCP, the energy input and range of the wavelength, retention time and flow rate is inspected [4]. However, there is no guarantee that the estimated energy dose in the appropriate Joules amount of UV-C is applied. This could be due to various reasons that affect all types of UV-C reactors (including UV-C milk processors), including bulb failure, diminished output over time, quartz sleeve fouling, improper exposure time and incorrect maintenance [5,6,7]. 

A review of the existing literature has revealed significant insights into the impact of light (visible light as well as UV) on proteins, lipids and vitamins, highlighting the changes in the fluorescence characteristics of these milk components.

**Figure 1:**
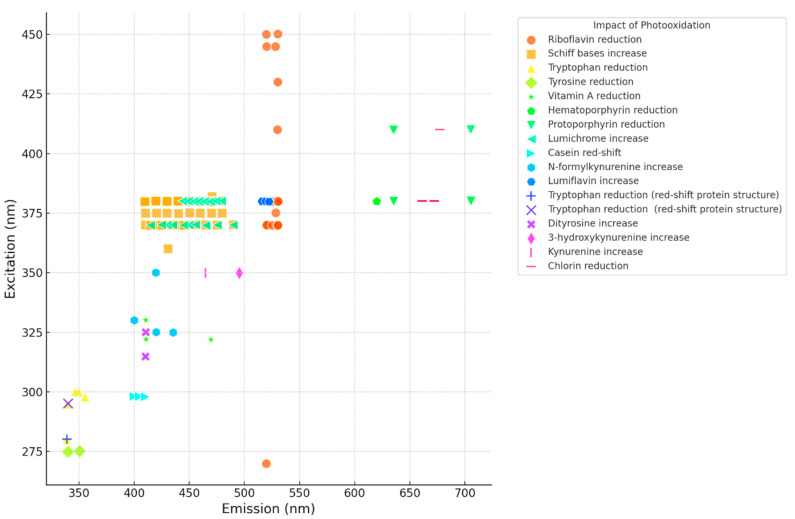
Scatter plot of the various photooxidation modifications in dairy products (excitation/emission nm) [8,9,10,11,12,13,14,15,16,17,18,19,20,21,22,23,24,25,26,27,28].

Figure 1 below is a summary of the impact of light (visible and UV light) on lipids, proteins and vitamins obtained from the reported literature [8,9,10,11,12,13,14,15,16,17,18,19,20,21,22,23,24,25,26,27,28]. It indicates the major changes in the fluorescence spectra (FLS) due to photooxidation from visible light (~400–760 nm) as well as UV-C (254 nm). The studies used include cheese, yogurt, butter, and, to a very limited extent, whole milk and isolated milk proteins. The fluorescence changes due to photooxidation share similarities throughout the different dairy products and the different wavelengths of light (vis and UV-C). For example, the increased intensity of the fluorescent Schiff base products (410–480 nm) as well as the reduction in intensity in the case of riboflavin emission (~520–530 nm) is observed in all products, the same as for protoporphyrin and chlorin compounds. 

These findings serve as a critical foundation for our ongoing research project. Our team has utilized these “quality biomarkers”, initially identified as indicators of light exposure during the storage of dairy products under light conditions (e.g., supermarket display), to establish a basis for our ongoing research project. This project aims to pinpoint the “fingerprint” changes in the fluorescence characteristics of milk when subjected to various doses of UV-C light during UV-C processing. Essentially, this research project is leveraging these “quality biomarkers” described by the studies mentioned in Figure 1 to assess the effectiveness of UV-C dose processing in milk. 

It is hypothesized that treating cow’s milk with UV-C light results in the photooxidation of lipids, proteins, and certain vitamins (like riboflavin), which in turn will cause unique (UV-C dose-dependent) changes to the fluorescence spectrum (FLS) of milk measured by fluorescence spectroscopy. These changes in FLS can serve as reliable predictors of the UV-C dosage and hence allow for the characterization of the UV-C processing as complete or incomplete, thereby ensuring the desired microbial inactivation. 

This assay is envisioned as an at-line (ultimately in-line), real-time monitoring, chemical-free, and environmentally friendly validation mechanism within the Hazard Analysis and Critical Control Points (HACCPs) framework, serving as a Critical Control Point (CCP). This methodological approach is analogous to the employment of the alkaline phosphatase test for the verification of pasteurization efficacy. There are no known quick verification tests for UV-C in milk, and there is no reference of it in the current literature. 

The rationale behind the mechanism of UV-C light affecting milk components and their fluorescence is still not very clear. No studies have investigated the impact of industrial UV-C processing units (controlled conditions and exposure to UV-C) on the fluorescence characteristics of milk components induced by UVC-photooxidation at 1045 J/L. The aim of this study is to communicate the preliminary findings on the application of right-angle fluorescence spectroscopy for identifying UV-C dose in milk, based on changes in the fluorescence spectrum of milk. The findings of our study represent the first attempt at such an analysis and require further refinement. Therefore, while the aim is to present the initial insights from this work, the readers are encouraged to focus on the conceptual framework and potential implications of this pioneering approach. 

## 2. Materials and Methods

### 2.1. Milk Sample Preparation and Processing Method

The milk (Charalmbides Christis LTD, Limassol, Cyprus) used was commercial pasteurized, homogenized, semi-skimmed bottled milk obtained from the local market. The chemical characteristics were provided by the manufacturer; it contained 1.5% fat, 3.3% protein, and 4.7% lactose, with a pH of 6.71. A total of 80 L of milk were processed (16 L every time for a total of 5 runs), with each sample dose being 250 mL. The UV-C reactor is a SurePure SP1 and the dosage applied in milk was estimated based on manufacturer’s instructions. Table 1 describes the experimental design. The milk temperature at the start of UV-C processing was 5 °C and at the end it was 9 °C; the temperature change was consistent across all five runs. The total processing time was 14 min and 3 s.

Dosage (J/L): This indicates the amount of UV-C exposure the sample receives, measured in J/L. Run time (adjusted for sample deduction): this shows the total time each sample spends in the UV-C reactor, adjusted for any time deductions due to sampling (note the increased frequency between 1000 and 1100 J/L). Volume in UV-C reactor after sample deduction (L): this is the volume of the sample left in the UV-C reactor after accounting for any deductions due to sampling. Biological replicates: The number of biological replicates for each dosage level. A biological replicate is an independent sample of the UV-C treatment, providing an estimate of variability between processes. Technical replicates per dose (FS): The number of technical replicates for each dose. Technical replicates are repeated measurements of the same sample to account for measurement variability. Total FS measurements per dose per chromophore: Each dosage level has five biological replicates, and for each biological replicate, three technical replicates are performed, resulting in a total of 15 FS measurements per dose for each chromophore. The samples were immediately stored in aluminum-covered 15 mL falcon tubes to prevent further photooxidation due to ambient light. Samples were stored in the refrigerator (−20 °C). The samples were thawed directly before measurement. The temperature of samples during fluorescence measurements was 5 °C. The sample 0 J/L was the control.

### 2.2. Fluorescence Measurements

Fluorescence experiments were conducted at a 90° angle using a FluoroLog FL3 spectrofluorometer from Horiba Jobin Yvon. The experiments utilized as variable wavelength excitation source, the monochromator-filtered output of a 450 W ozone-free xenon lamp, as described by [29]. The measurements were carried out as shown in Table 2 below. A total of 3 mL milk were placed in a 3.5 mL UV (200–2500 nm) Ossila quartz cuvette (1 × 1 cm, path length 10 mm). The fluorescence signal was corrected for both intensity fluctuations of the xenon lamp excitation as well as the spectral response of the grating and the visible PMT (Horiba TBX module, 250–950 nm) detector used. 

The chromophore areas described in Table 2 were chosen based on the literature used to develop Figure 1 [8,9,10,11,12,13,14,15,16,17,18,19,20,21,22,23,24,25,26,27,28].

### 2.3. Statistical Analysis

Data analysis was carried out in OriginPro 2023. The data meet the assumptions required for a one-way ANOVA; Shapiro–Wilk test and Levene’s test (*p*-value > 0.05). Tukey’s post hoc test was used to compare all possible pairs of group means after an ANOVA has found a significant difference [30].

## 3. Results

**Figure 2:**
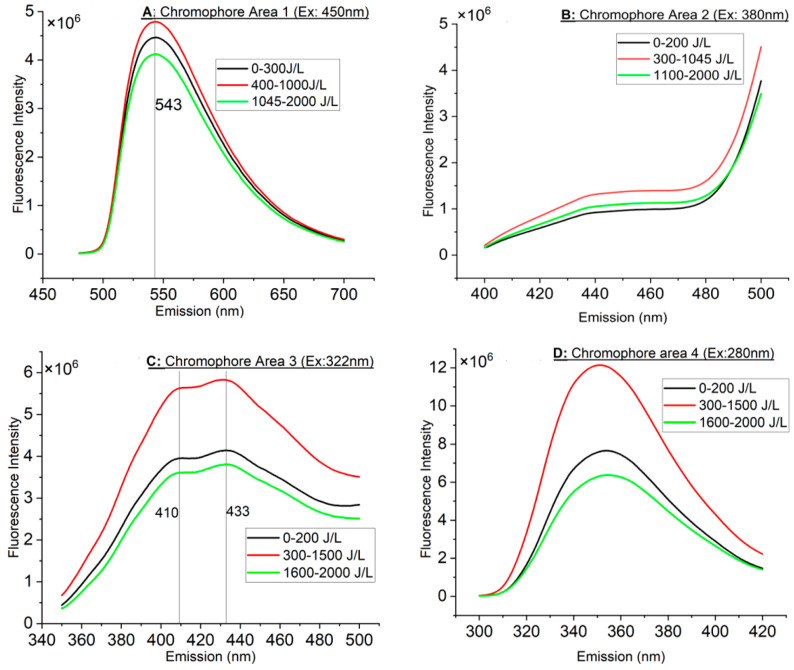
(**A**,**B**) Averaged fluorescence spectra (FLS) of CA 1 and 2. Vertical lines indicate emission peak. (**C**,**D**) Averaged fluorescence spectra (FLS) of CA 3 and 4. Vertical lines indicate emission peak.

The maximum emission peak of the fluorescence spectrum of the milk upon blue photoexcitation at 450 nm is 543 nm, as shown in Figure 2A. UV-A photoexcitation at 380 nm results in a broad fluorescence extending from approx. 430 nm to over 470 nm, as observed in Figure 2B (beyond 470 nm riboflavin’s peak interferes). For shorter UV-A excitation at 322 nm, two peaks were noticed in Figure 2C at 410 nm and 433 nm, while the employment of deep UV-B excitation at 280 nm results in emission peaking at approximately 355 nm in Figure 2D. The strongest emitters were in decreasing order as follows: CA 4, 3, 1 and finally 2 (Figure 2A–D). 

For all CAs, three major groups of FLS were identified, as shown in Figure 2A–D. In Figure 2A, the FI increased at about 400 J/L and remained high up to approximately 1000 J/L. At approximately 1045 J/L and beyond, the intensity dropped below the group 0–300 J/L. In comparison, in CA 2 (Figure 2B), beyond 1045 J/L, the FI remained higher than the group 0–200 J/L. In Figure 2C,D, the increased FI was extended from 300 J/L to 1500 J/L instead of ~1045 J/L (as in CA 1 and 2). Beyond 1500 J/L, the FI dropped below that of the 0–200 J/L group.

**Figure 3:**
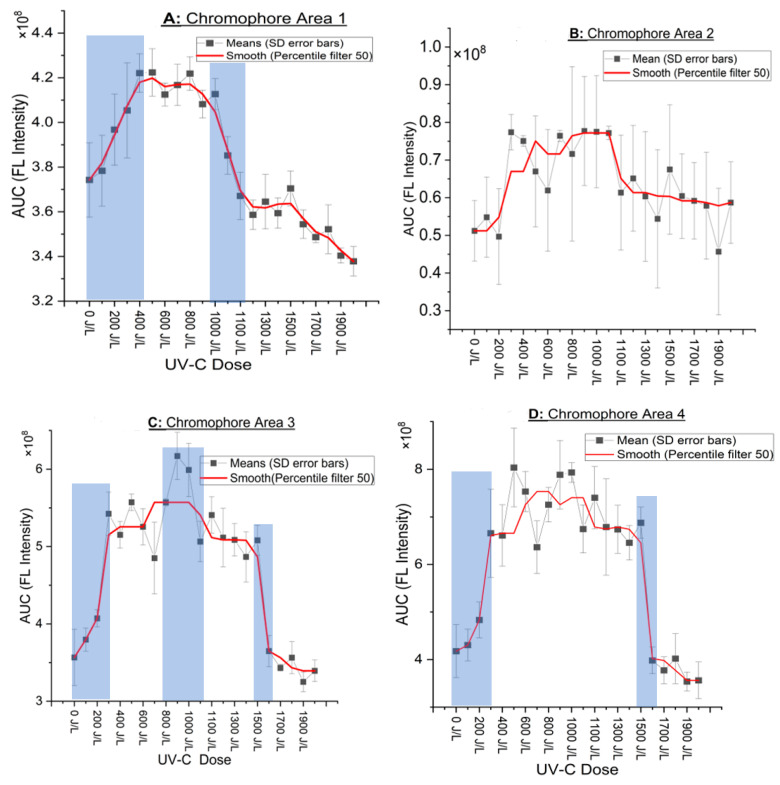
(**A**–**D**) Plots of the integrated fluorescence quantified by the area under the curve (AUC) at each UV-C dose with standard deviation error bars and a red-colored smoothed curve (percentile filter 50) for easier presentation. *Y*-axis is the AUC intensity and *X*-axis is the UV-C dose applied. The shadowed areas indicate the areas where the major, statistically significant changes occurred.

The observations described in the 4 CAs in Figure 2A–D related to the fluorescence intensity variation versus dose (J/L) are better resolved in Figure 3A–D, where detailed diagrams of the UV-C dose (J/L) and the evolution of integrated emission area (AUC) are provided. Figure 3A–D illustrate, for example, the general trend of a steep increase in AUC for all CAs above 200–300 J/L and a subsequent steep drop in AUC at doses of 1000–1500 J/L. The shadowed areas in Figure 3A–D indicate the areas where the major, statistically significant changes occurred (described in Table 3).

In CA 1 (Figure 3A), there are two plateaus, one at 400–1000 J/L and the second at 1100–1500 J/L, each followed by a drop in intensity. In CA 3 and 4 (Figure 3C,D), there is one plateau ranging from 300 J/L to 1500 J/L followed by drop in intensity. In CA 3 (Figure 3C), in the plateau that extends from 300 to 1500 J/L, there is a peak that emerges around 900–1000 J/L, which significantly differs from the rest points.

**Figure A1:**
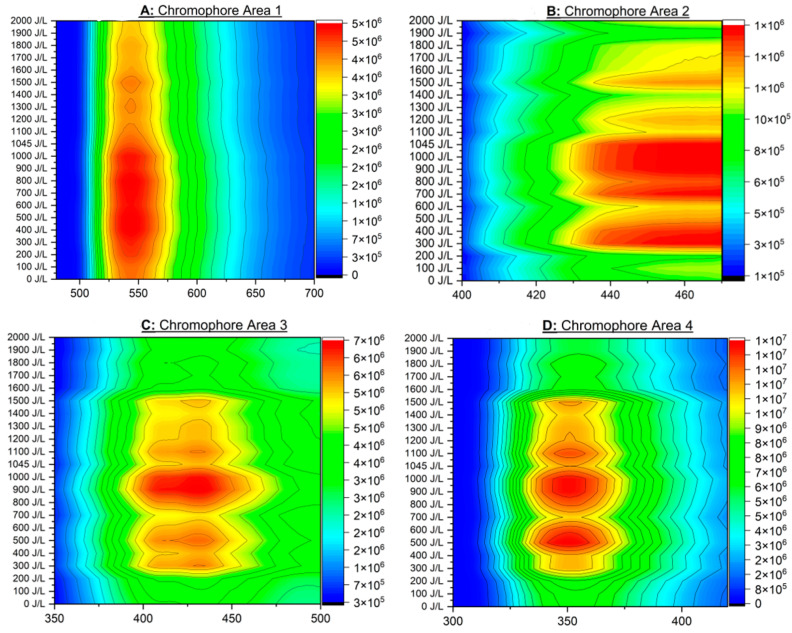
(**A**–**D**) Contour maps for chromophores at the excitation–emission parameters described in Table 2. *Y*-axis is the UV-C dose applied and *X*-axis is the emission. These contour maps were developed using the same dataset as for Figure 2A–D.

There was an increased discrepancy (high standard deviation) in CA 2 (Figure 3B) between samples, which suggests the possible short lifespan of these chromophores. Nonetheless, after applying a 50th percentile “median” filter on the graph, the smoothed curve (red) indicates a similar pattern as in the case of the other CA described earlier, i.e., an increase in FI initially, followed by a plateau and then a steep drop near 1000–1100 J/L. No statistical analysis was carried out due to the high SD. The future experiments will aim for immediate fluorescence scan, considering their instability. In addition to the four CA described above, the tetrapyrolles (protoporhyrrins and hematoporphyrins) were also investigated; however, there were no peaks, and hence they will not be discussed further. The statistically significant points described in Figure 3A–D are shown in Table 3 below. For easier interpretation of Figure 2A–D and Figure 3A–D, contour maps (Figure A1A–D in Appendix A) are used to highlight the key points more clearly. 

## 4. Discussion

### 4.1. Chromophore Area 1 (CA 1)

The excitation–emission settings described in Table 2 for CA 1 correspond to riboflavin. Riboflavin is a fluorescent compound with three excitation maxima, one of which is 450 nm and is reported to emit at 520–535 nm in milk and other dairy products [8,10,17,20]. Indeed, the as-detected fluorescence of riboflavin in the spectrofluorometer setup used is at 534 nm, which agrees with the literature. However, when the calibration correction for the grating and detector spectral response is applied, the emission peak shifts to 543 nm, as seen in Figure 2A. As this calibration has been independently confirmed to perform well in reference samples with a known emission and has been reliably applied many times, it was considered that 543 nm was a better estimate for the peak fluorescence of riboflavin contained in the specific type of milk used. Nonetheless, this small deviation is unimportant for this study, since the focus is placed on the fluorescence intensity changes rather than potential spectral shifts in comparison with the control 0 J/L sample.

There are no studies on the impact of industrial UV-C reactors and the changes on the fluorescence of milk constituents. The majority of studies assessing the impact of light on riboflavin in milk and dairy products are concerned with visible light from the quality aspect during storage in, e.g., supermarkets displays [8,10,12,13,14,15,17,18,19,20,21,28]. Only one study investigated the impact of UV-C on riboflavin in milk [22]. However, the exposure time was 90 min under 2 × 15 W UV-lamps in a stationary, non-agitated condition (placed in quartz cuvette) [22]. In comparison, in this study, a tightly controlled UV-C exposure in an industrial UV-C reactor was followed to achieve the allowed 1045 J/L (EFSA) [4] under controlled flow and turbulence. Nonetheless, all studies report a drop in riboflavin’s FI in milk exposed to UV-C or visible light [8,10,12,13,14,15,17,18,19,20,21,22,28]. 

Surprisingly, in this study, initially, a steep and significant increase in the FI of riboflavin was observed, as described in Table 3 and Figure 2A and Figure 3A. This is a very interesting and new observation. A possible explanation behind this is given in this paragraph. Perhaps UV-C induces the release of bound riboflavin; this can be understood based on the following two points. The first being that, according to the study by Leviton A. and Pallansch M.J., riboflavin in milk binds predominantly to α-casein and β-casein at the phenoxyl residues. The calcium caseinate–phosphate complex in milk also binds riboflavin, but this binding is equivalent to that of its individual protein components, suggesting that the micellar structure of the complex does not provide any additional binding capacity beyond the individual proteins. It is noted that riboflavin binds to these proteins with a relatively low affinity [30]. Also, riboflavin is non-specifically bound to the protein elements found in the milk fat globule membrane [31]. The second point is that riboflavin is the key building block for its co-enzymatic forms Flavin Adenine Dinucleotide (FAD) and Flavin Mononucleotide (FMN). The excitation and emission characteristics of FMN are similar to riboflavin (i.e., excitation: 450 nm and emission: 525 nm) [32]. The charged flavin species like FMN and FAD are readily bound to calcium-rich micelles in milk [31]. Consequently, considering the fact that riboflavin and its co-enzymatic forms are associated with proteins [31], the initial increase in the FI of riboflavin is attributed to the possibility that UV-C induced the release of riboflavin from the proteins, contributing to higher FI. Another possibility is that the proteins to which Riboflavin is bound change configuration (unfolding or denaturation), exposing riboflavin, facilitating in this way better excitation and emission. 

The FI of riboflavin, after 1100 J/L dropped below the FI of the control sample (0 J/L), followed a reducing trend, but the FI was significantly lower than the control (0 J/L) only at 1700 J/L (see Figure 3A). Riboflavin is sensitive to light, particularly in the 200 to 500 nm range. Riboflavin serves dual roles in the context of photochemical reactions. Not only is its an efficient photosensitizer capable of generating singlet oxygen upon exposure to light, but it also acts as a reactive substrate for singlet oxygen [33,34]. Therefore, that indicates that riboflavin and other photosensitizers are damaged not only due to their own excitation but are also directly attacked by singlet oxygen, like in the case of lipids and proteins. In other words, when photosensitizers are involved in photoreactions, whether as part of type I reactions or through interactions with singlet oxygen in type II reactions, they undergo degradation, a process known as photobleaching [8]. This might explain why between 1100 and 2000 J/L (Figure 3A) there is a constant reduction in FI of riboflavin with increasing UV-C dose.

The degradation of riboflavin is depended on the amount of fat in the milk; the higher the fat content of milk, the lesser the degradation [35], possibly due to the light scattering properties of milk fat [36]. Considering that the milk used in this study was semi-skimmed, in theory, milk with lower fat would have shown a faster decrease in FI, and vice versa. This will be examined in future experiments.

In summary, it was hypothesized that the initial increase in the FI of riboflavin is due to changes in the protein–riboflavin structural relationship or protein structure. UV-C light more probably influences the binding of riboflavin on such complexes, facilitating its release, which results in higher fluorescence intensities. At higher UV-C doses, the drop in FI is possibly attributed to the photobleaching of riboflavin.

### 4.2. Chromophore Area 2 (CA 2)

The CA 2 (Table 2) corresponds to tertiary oxidation products. A study showed that UV-C treated milk (3.5% fat) exhibited increased FI at 410–480 nm, which corresponds to the formation of fluorescent tertiary oxidation products [22]: conjugated Schiff bases created through the reaction between amino groups and secondary lipid oxidation products (aldehydes) [37]. The same observation occurs when milk and other dairy products are exposed to visible light [10,11,12,19,28]. Kikugawa and Bebu described in detail the chromophores responsible for that broad peak mentioned in the literature. Some of these generated fluorophores have an excitation maxima at 350–400 nm and emission maxima at 450–470 nm [37,38]. This could possibly explain some of the fluorophores that are present in the emission wavelength range of 435 to 470 nm in our study and other studies [10,11,15,17,28].

Nonetheless, there is another chromophore that emits at ~444–479 nm when excited at 370–380 nm; it is called lumichrome [9,14,15,18,28]. UV-C light affects riboflavin in milk, causing its photodegradation due to photochemical decomposition and photosensitization [22]. Lumichrome is one of the two biproducts of riboflavin degradation and exhibits strong fluorescence as well [15]. 

The obtained FI of CA 2 was strong, but there was a large discrepancy between samples (compared to the other three CAs) indicating a possible instability of chromophores in that excitation–emission range. Indeed, Schiff bases are unstable, and it is highlighted that a more detailed and rigorous analysis is essential [37]. Also, it should be noted that lumichrome is also a photosensitizer, meaning that it generates singlet oxygen which eventually becomes degraded by it (photosensitized degradation) [33]. These, collectively, could partly explain the higher standard deviation in CA 2 compared to the other CAs.

The overarching takeaway is the consistent observation across various studies that lipid oxidation in food products leads to the formation of fluorescent compounds, particularly at an emission range of 400–500 nm. This fluorescence is attributed to complex reactions involving lipid oxidation products, proteins, and amino acids, with Schiff base adducts playing a significant role in this process. These findings contribute to our understanding of lipid oxidation in food science, offering potential methods for detecting and studying oxidative changes in food products.

Perhaps future experiments would consider the instability of chromophores in this excitation–emission area, and ideally analyze the samples immediately after the UV-C treatment of milk. 

### 4.3. Chromophore Area 3 (CA 3)

As shown in Table 4, vitamin A and dityrosine have similar excitation and emission characteristics and therefore, in Figure 2C, the emission peak at 410 is possibly a combination of vitamin A and dityrosine, whereas the peak at 433 nm is N-formylkynurenine (NFK).

Vitamin A typically has its highest excitation and emission at 322–330 and 410 nm, respectively [10,11,22]. However, UV induces the geometric isomerization of vitamin A, producing the 5,6 epoxyretinol which excites at 350 nm and emits at 470 nm [22,39]. The future experiments will include excitation at 350 nm and an emission range including 470 nm to see if the drop in Vitamin A intensity is correlated with an increase in FI of 5.6 epoxyretinol.

Both types of light (visible and UV-C) induce oxidative effects, with UV-C light causing more significant changes, including increased protein carbonyl formation and higher levels of dityrosine, which forms through the cross-linking of tyrosine residues [40]. Neutral radicals produced through photoionization can engage in radical–radical coupling reactions, leading to the formation of dimeric structures. The coupling of tyrosyl radicals specifically results in the creation of dityrosine and isodityrosine [41,42,43,44]. While dityrosine and isodityrosine are formed simultaneously, isodityrosine does not have fluorescent properties like dityrosine displays. Dityrosine is considered the principal product of tyrosine oxidation [40,43,45]. Based on this information, and the fact that dityrosine has an excitation at 315–325 nm and an emission maximum at 410 nm (Table 4), it is very likely that the peak in Figure 2C at 410 nm could be related to dityrosine, in addition to vitamin A, as described above. 

N-formylkynurenine (NFK) is a product of tryptophan oxidation by singlet oxygen under light conditions [16,23,24,46]. In addition to NFK, kynurenine is also a fluorescent by-product of tryptophan’s photooxidation, but not all studies mention kynurenine because its fluorescence is weak (with an emission maximum at 480 nm and excitation at 365 nm). NFK is also a weak emitter of fluorescence but stronger than kynurenine (emission maximum is 434 nm on an excitation at 325 nm) [47,48]. The presence of NFK indicates potential protein fragmentation [27]. Tryptophan naturally absorbs UV light within the 240–310 nm wavelength range and gradually converts it into NFK [49,50]. In this study, the FI of NFK, instead of increasing with increasing UV-C dose, undergoes a steep drop at 1500 J/L. A possible explanation is that tryptophan and its metabolites, NFK, kynurenine and 3-hydroxykynurenine, are photosensitizers [51,52,53], and as explained, photodegradation or photobleaching is a general characteristic of photosensitizers [8,54,55]. That might explain the decrease in FI after 1500 J/L. Another possible contributor might be that NFK is non-enzymatically converted to kynurenine [49,50].

### 4.4. Chromophore Area 4 (CA 4)

CA 4 excitation and emission settings correspond to tryptophan (excitation: 280; emission: 350). In addition to tryptophan, tyrosine can also serve as an intrinsic fluorescent probe within proteins. Tyrosine excites at 275–280 nm and emits at 302–305 [56], even though several authors mentioned that tyrosine emits at 350 [10,25]. Even if tyrosine could emit at 350 nm, this would be insignificant because tyrosine has a significantly lower extinction coefficient than tryptophan, and its fluorescence emission is usually overshadowed by that of tryptophan. Consequently, tyrosine fluorescence is typically utilized only in proteins that lack tryptophan [56,57]. Moreover, 3,4-dihydroxyphenylalanine (DOPA) is a tyrosine oxidation by-product that can be formed through the reaction of oxygen with tyrosine free radicals that are generated by UV or ionizing radiation [58,59,60]. Like tyrosine, DOPA in aqueous solution is known to show fluorescence in the near-ultraviolet (UV) spectral region when it is excited at wavelengths below 300 nm [58]. DOPA has a maximum excitation wavelength (λ max) at 280 nm and a maximum emission wavelength (λ max) at 320 nm. This differentiates DOPA from dityrosine, which has excitation (315–325 nm) and an emission at 410 nm [60]. Interestingly, both DOPA and dityrosine differ from their parent tyrosine, which has a maximum excitation wavelength (λ max) at 275 nm and emission at 340–350 nm [10,25]. Nonetheless, as shown in Figure 2D, whether DOPA was generated or not is not known, since there is no emission peak at 320 nm at 280 nm excitation, indicating that DOPA is a weak emitter or that UV-C photooxidation favors the generation of dityrosine (as described in CA 3) rather than DOPA. Therefore, the fluorescence observed in CA 4 is considered to be attributed to tryptophan.

From the chromophores that were investigated in this study, tryptophan had the highest fluorescence intensities (see Figure 2A–D) with the sharper increases and decreases in FI induced by UV-C. As shown in Figure 3D and Table 3, the FI significantly increases by 60% at 300 J/L and remained at a plateau between 300 and 1500 J/L and then dropped significantly by 70% after 1500 J/L. After that point (<1500 J/L), the spectra followed a reducing trend with increasing UV-C dose. 

An increase in the FI of tryptophan was described in another study, which reported that after prolonged exposure to UV light there was increase in emission from tryptophan in α-lactalbumin and β-lactoglobulin at 355 nm, describing that tryptophan might have become more exposed due to protein unfolding [16]. Another study described the above phenomenon in detail with bovine β-Lactoglobulin (β-Lg). Tryptophan-61 is close to the disulfide bridge between Cys66 and Cys160. This disulfide bridge is thought to effectively quench tryptophan fluorescence [61]. Therefore, the higher FI of tryptophan in our study could have also been attributed to the cleavage (due to UV-C photooxidation) of the disulfide bridge [24] which effectively quenches tryptophan fluorescence [61]. 

In our study, at >1500 J/L (see Figure 3D), there was a significant reduction in FI. Two studies also described a decrease in the FI of β-lactoglobulin, suggesting the association or polymerization of proteins (dimer formation) [61,62]. Indeed, there is a large number of research papers which show that the photooxidation of milk causes protein polymerization, oligomerization or aggregation. These changes are observed in experimental designs with either visible, UV-B or UV-C light [16,23,26,27,40]. Future studies will include proteomics (e.g., electrophoresis and others) to verify changes in the molecular mass of proteins and changes in the fluorescence characteristics of milk. In addition to protein association, the reduced fluorescence intensity at >1500 J/L might be attributed to the loss of tryptophan due to photooxidative degeneration in the presence of riboflavin [16].

Overall, based on the information provided above, the changes in the fluorescence spectra of CA 4 are possibly attributed to changes in the structure of proteins, which ultimately alters the microenvironment of tryptophan. Also, the fact that photooxidation can generate new disulfide bridges [40], which are able to quench the emission of tryptophan [61], means that they are, therefore, also possible contributors of the reduced emission after 1500 J/L.

### 4.5. Future Research

The robustness of this method of analysis could possibly be affected by the inherent photooxidative stability of milk. This hypothesis is based on the findings from different studies. For example, riboflavin is the primary photosensitizer of milk, and in a study by Shiota et al., the riboflavin content was the major influencer of the photooxidative stability of ice cream [63]. In addition, milk naturally scatters light, and this is attributed to milk fat globules and casein micelles [64]. It is stated that the effectiveness of UV-C radiation in milk is affected by the presence of fats and proteins by acting as “natural sunscreens”, which reduce the penetration of UV-C light in milk [65]. Also, whether the milk is homogenized or not affects light scattering, due to modifications in the fat globule membrane and the decrease in free casein micelles [64]. Finally, the overall oxidative stability of milk varies due to different lactation periods, breeds, and milking regimes [66]. 

To conclude, since the changes in interest are products of photooxidation, the factors that affect photooxidation might impact the accuracy of this analytical method, leading to potentially false positive and false negative results. Therefore, incorporating variability into the dataset, by including these diverse milk samples with varied oxidative stability (e.g., homogenization, different lactation periods, fat content, etc.), allows for developing a stronger training dataset for chemometrics analysis and machine learning. The goal of our method of analysis is to distinguish between different UV-C doses across all these types of milk with different oxidative stabilities, hence including this variability in the future experiments is crucial. 

Lastly, in future experiments, the changes in the fluorescence of the other chromophores described in Figure 1 above will also be investigated as potential biomarkers. Furthermore, several types of analyses will be conducted in order to determine chemical and structural changes in proteins induced by photooxidation during the UV-C treatment to help explain the observed changes in the fluorescence spectrum of milk. For example, an analysis for DNPH protein carbonyls [23,67], the development of intra- and inter-molecular dityrosine bonds [27], thiol groups (total sulfhydryl content) using the Ellman method [68,69], dynamic light scattering (DLS) [40], electrophoretic mobility (e.g., SDS-PAGE) [23], and variations in surface hydrophobicity [70]. Other chemical analyses, to determine the content of vitamins like A and riboflavin as well as to assess the degree of lipid oxidation, will be carried out. 

### 4.6. Future Spectroscopic Setup Improvements

No rapid tests or literature references exist for UV-C verification in milk. In this preliminary study, right-angle fluorescence spectroscopy was employed. This kind of configuration is known to be disadvantageous in milk due to its high-absorbance, inner filter effect [61]. However, the results obtained in our study indicate that it is possible to use a right angle for the purpose of monitoring the spectral evolution during UV-C processing. In addition, as already mentioned, the emissions obtained are in accordance with the literature. Moreover, a study compared the fluorescence emission spectra of cow and buffalo milk, identifying key differences in their fluorescence at specific band positions (382 nm, 440 nm, 505 nm, and 525 nm) using both classical (right angle) and front-face fluorescence setups. The results from this study suggested that both right-angle and front-face fluorescence setups can effectively differentiate cow and buffalo milk, serving as a fingerprint for identification [71]. Similarly, in this study, preliminary verification tests revealed that front-face fluorescence measurements taken at a 45° angle yielded results comparable to those obtained using right-angle spectroscopy. A more systematic comparative study that will probe in detail the impact of optical geometry, excitation area and power, and the use of free space versus optical fiber communication is under way, and will be the subject of future work.

Furthermore, front-face fluorescence spectroscopy (FFFS) will be investigated, in the near future, as studies [14,21,72,73,74,75,76,77,78,79,80] have shown the applicability and sensitivity of this method to study changes in the fluorescence spectra of milk components due to photooxidation. In addition to fluorescence spectroscopy, the future research projects will include reflectance and Raman (e.g., the coherent backscattering of light), which has been shown to be an ultrasensitive and quick method of detection of milk protein denaturation and the oxidation in edible proteins and lipids [81,82,83,84]. Hence, future studies will investigate the performance of three different optical techniques, namely front-face fluorescence (FFFS), front-face reflectance (FFRES) and front-face Raman (FFRAS) spectroscopies. Our ultimate goal is to create a test that will be in situ, real-time, non-invasive, and free of chemicals and consumables for eco-friendly, real-time monitoring.

## 5. Conclusions

This study represents a pioneering effort in using intrinsic chromophores and fluorescence spectroscopy to determine UV-C doses in UV-C treated milk. The preliminary findings demonstrate the potential of this innovative approach, highlighting significant fluorescence changes in chromophores correlating with UV-C doses. Future studies will focus on fine-tuning this method to ensure its reliability and applicability across various conditions and milk types by pinpointing the “fingerprint” changes in the fluorescence characteristics of the chromophores described earlier (i.e., in Figure 1). The promising results lay the groundwork for more comprehensive investigations to fully establish fluorescence spectroscopy as a viable biomarker tool for UV-C dose verification in the dairy industry. It should be further noted that the development of such a test (ideally in the processing line) should prove of great benefit to the food industry, given that UV-C treatment is used as an alternative to heat treatments, i.e., those that use juice or beer, to achieve a reduction in pathogens and render the product safe for human consumption.

## Figures and Tables

**Table 1:** Time of sampling per UV-C dose and replicates. Run time (adjusted for sample deduction). Volume in UV-C reactor after sample deduction.

Dosage	Run Time	Volume	Total Biological Replicates	Technical Replicates per Sample
0 J/L (Control)	00 min-00 s	16.00 L	5	3
100 J/L	01 min-02 s	15.75 L	5	3
200 J/L	02 min-02 s	15.50 L	5	3
300 J/L	02 min-59 s	15.25 L	5	3
400 J/L	03 min-55 s	15.00 L	5	3
500 J/L	04 min-49 s	14.75 L	5	3
600 J/L	05 min-41 s	14.50 L	5	3
700 J/L	06 min-31 s	14.25 L	5	3
800 J/L	07 min-19 s	14.00 L	5	3
900 J/L	08 min-05 s	13.75 L	5	3
1000 J/L	08 min-49 s	13.50 L	5	3
1045 J/L	09 min-03 s	13.25 L	5	3
1100 J/L	09 min-21 s	13.00 L	5	3
1200 J/L	10 min-00 s	12.75 L	5	3
1300 J/L	10 min-37 s	12.50 L	5	3
1400 J/L	11 min-13 s	12.25 L	5	3
1500 J/L	11 min-46 s	12.00 L	5	3
1600 J/L	12 min-17 s	11.75 L	5	3
1700 J/L	12 min-47 s	11.50 L	5	3
1800 J/L	13 min-14 s	11.25 L	5	3
1900 J/L	13 min-40 s	11.00 L	5	3
2000 J/L	14 min-03 s	10.75 L	5	3

**Table 2:** This table describes the parameters in each of the fluorescence locations (chromophores).

Chromophores Area	Ex (nm)	Em (nm)	Ex Bandwith (nm)	Em Bandwith (nm)	Filter at Reader	Increment	Integration Time
CA 1	450	480–700	3.00	3.00	LP495 nm	1 nm	0.1 s
CA 2	380	400–480	3.00	4.98	LP400 nm	1 nm	0.1 s
CA 3	322	350–500	3.00	3.00	n/a	1 nm	0.1 s
CA 4	280	300–420	3.00	3.00	n/a	1 nm	0.1 s

**Table 3:** Statistical analysis. One-way ANOVA, Tukey’s post hoc test.

Chromophore Area	UV-C Dose (J/L)	FI	Difference in FI (Statistical Significance)
CA 1	0–400	13% increase	<0.0001
	1000–1045	10% decrease	<0.05
CA 3	0–300	52% increase	<0.0001
	800–900	17% increase	<0.05
	1000–1045	25% decrease	<0.0001
	1500–1600	40% decrease	<0.0001
CA 4	0–300	60% increase	<0.0001
	1500–1600	70% decrease	<0.0001

**Table 4:** Excitation–emission characteristics of chromophores.

Chromophore	Excitation (nm)	Emission (nm)	Product (Light)	Reference
N-formylkynurenine	330	400	Isolated milk proteins (Visible light)	[16]
N-formylkynurenine	325	435	Milk (UV-C)	[27]
N-formylkynurenine	325	420	Isolated milk proteins (UV-C)	[23]
N-formylkynurenine	350	420	Isolated milk proteins (UV-C)	[23]
Dityrosine	325	410	Milk (UV-C)	[27]
Dityrosine	315	410	Isolated milk proteins (UV-C)	[23]
Vitamin A	322	411	Cheese (Visible light)	[11]
Vitamin A	330	410	Cheese (Visible light)	[10]
Vitamin A	322	410	Milk (UV-C)	[22]

## Data Availability

The original contributions presented in the study are included in the article, further inquiries can be directed to the corresponding author.

## Appendix A

The contour maps reveal how FI changes across the different UV-C doses. At low UV-C doses (near the bottom of the *y*-axis), the FI is relatively low, as indicated by the blue and green colors. As the UV-C dose increases, the FI increases, shown by the transition to yellow and then red colors at higher doses.

In CA 1 (Figure A1A), there is a prominent, continuous red region which spans approximately the UV-C doses 300–1000 J/L. This area represents the highest values on the plot. In CA 2, even though not analyzed statistically, it indicates that the emission range 430–470 nm shows two red regions from 300 nm to 1045 nm.

In CA 3 and 4 (Figure A1C,D), the highest fluorescence intensities are seen sporadically in the following UV-C dose ranges: 300–600 J/L, 800–1000 J/L, and less intensely around 1100 J/L and 1500 J/L (beyond that, the FI drops significantly).
